# Pericytes and Diabetic Microangiopathies: Tissue Resident Mesenchymal Stem Cells with High Plasticity and Regenerative Capacity

**DOI:** 10.3390/ijms26115333

**Published:** 2025-06-01

**Authors:** Zeinab Shirbaghaee, Christine M. Sorenson, Nader Sheibani

**Affiliations:** 1 Department of Ophthalmology and Visual Sciences, University of Wisconsin School of Medicine and Public Health, Madison, WI 53705, USA; shirbaghaee@wisc.edu; 2 McPherson Eye Research Institute, University of Wisconsin School of Medicine and Public Health, Madison, WI 53705, USA; cmsorenson@pediatrics.wisc.edu; 3 Department of Pediatrics, University of Wisconsin School of Medicine and Public Health, Madison, WI 53705, USA; 4 Department of Cell and Regenerative Biology, University of Wisconsin School of Medicine and Public Health, Madison, WI 53705, USA

**Keywords:** diabetes complications, metabolic activities, perivascular supporting cells, blood barriers, extracellular vesicles, central nervous system, extracellular vesicles

## Abstract

Pericytes (PCs), a heterogeneous population of perivascular supporting cells, are critical regulators of vascular stability, angiogenesis, and blood–tissue barrier integrity. Increasing evidence highlights their active role in the pathophysiology of diabetic microangiopathies, including those affecting the retina, kidney, brain, heart, and peripheral nerves. In diabetes, hyperglycemia-induced PC dysfunction is a major contributor to vascular degeneration, impaired tissue repair, and disease progression across multiple organs. Pericytes also share many characteristics with mesenchymal stem cells (MSCs). They exhibit regenerative capacity, immunomodulatory activities, and multipotent capacities. This review explores the emerging role of PCs as tissue resident MSCs, emphasizing their pathophysiological involvement in diabetes complications, and their potential for utilization in regenerative medicine. We also discuss advances in PC-based therapies, tissue engineering strategies, and clinical applications. Thus, PCs are positioned as promising targets for therapeutic intervention and vascular tissue regeneration.

## 1. Introduction

A heterogeneous cell population of perivascular supporting cells, known as smooth muscle cells/pericytes (SMCs/PCs) or collectively mural cells, surround endothelial cells (ECs) in capillaries, arterioles, and venules of the vasculature circulatory system. PCs typically reside within the basement membrane on the abluminal surface of most capillaries, and in some larger blood vessels, which are shared with ECs [1,2]. The vascular basement membrane is composed of extracellular matrix (ECM) proteins produced and shared by both ECs and PCs. The basement membrane connects these cells and provides structural support and heterotypic cell–cell communications through protrusions (pegs) inserted into EC invaginations (sockets) at occasional interruptions in the basement membrane [3]. Previously, it was impossible to distinguish PCs from other perivascular cells. This gave rise to the idea that PCs are basically inactive supporting cells whose only physiological role is vascular stability [4].

Over the past few decades, the functional characteristics of PCs have considerably expanded [5]. Until the end of the 20th century, PCs were mainly identified based on their anatomical location and morphology. PCs have long processes surrounding the blood vessel walls, and are widely dispersed in all tissues [6]. They wrap around ECs and communicate with them along the length of blood vessels via paracrine signaling and physical contacts [7]. Their interaction with the glial and neuronal cells of the central nervous system (CNS) establishes the neurovascular unit (NVU) that is vital to the proper health and function of CNS tissues.

PCs are known to play a significant role in blood flow modulation. The ratio of ECs to PCs is different in the vasculature of various tissues, and significantly higher in the blood vessels of the CNS, including the retina and brain, compared to other tissues. Given the existence and significance of a blood barrier, and its importance in the integrity and function of the CNS vasculature, the high density of PCs in this vasculature could play a significant role. Researchers are increasingly interested in the biology of PCs, focusing on their role in vascular homeostasis and disease, and expanding our understanding of their role in pathophysiological processes, including fibrosis [8]. Recent studies have identified PCs as a key mediator of fibrosis in many tissues, including the eyes [9].

PCs normally contribute to the maintenance of vascular rheology by regulating blood circulation, vascular structural stability, vascular permeability, angiogenesis, and the establishment of the blood–brain/blood–retina barrier (BBB/BRB) [10,11,12]. PCs cooperate with ECs, astrocytes, and neurons in the CNS [13,14], and regulate the integrity of the vascular barrier characteristics [15]. This is accomplished through EC- and PC-derived products, including the platelet-derived growth factor-B (PDGFB)/PDGF receptor-beta (PDGFRβ) signaling pathway, which is vital for PC recruitment, barrier development, and the maturation of blood vessels [16]. Inappropriate signals, defective metabolism, and pathological circumstances, such as inflammatory and oxidative stress, impede communication between ECs and PCs resulting in the breakdown of the BRB and other microangiopathies [17]. PC loss in diabetic retinopathy (DR), age-related macular degeneration (AMD), and uveitis causes the BRB breakdown and the infiltration of inflammatory cells, enhancing the progression of the pathological processes in these retinopathies [18,19].

PCs also play a crucial role in immunomodulatory and phagocytic activities [20], such as regulating the stimulation of lymphocytes in the retina [21], appealing innate leukocytes to withdraw by sprouting blood vessels in the skin [22], and removing detrimental cellular byproducts in the brain and retina through direct phagocytic activity [23]. They can also acquire tissue-specific roles through dedifferentiation to other cell types [5]. PCs exhibit diverse morphologies and marker profiles, making their immunocytochemical characterization challenging due to their phenotype’s reliance on vascular bed, resident tissue, activity conditions, and their differentiation state [24,25,26]. PCs can differentiate into a variety of cell types due to their multipotent differentiation capability. Thus, these cells are promising targets for tissue repair and therapeutic techniques in regenerative medicine [24,27]. In various tissues, PCs can act as resident mesenchymal stem cells (MSCs) [28], producing different cell types, while regulating the activity of other stem cells similar to hematopoietic stem cells in their niches [29,30,31].

PCs exhibit various MSC attributes stemming from the embryonic mesodermal layer with comparable surface markers and differentiation potential [32,33]. Previous studies have demonstrated that cultured human adipose-derived MSCs may spontaneously manifest a PC-like phenotype [34,35]; nevertheless, contingent on culture conditions, spontaneous differentiation into ECs has also been documented [36]. PCs in the CNS are neural crest-derived but share many characteristics with PCs in the vascular beds of other tissues, including their MSC characteristics. MSCs exhibit potent immunoregulatory capabilities which can hinder deleterious inflammatory reactions in the ailing retina. The manifold production of growth and neurotrophic factors by MSCs bolsters the survival and expansion of the retinal cells. Furthermore, the anti-apoptotic characteristics of MSCs enable them to safeguard retinal cells, potentially facilitating the regeneration of the ailing retina by virtue of their ability to differentiate into an array of cell types, including various retinal cell types. These qualities, taken together, highlight the potential of MSCs for treating damaged retinas. The most recent results of several experimental investigations conducted in diverse preclinical models have strengthened this viewpoint [37].

MSCs release diverse categories of extracellular vesicles (EVs). These particles selectively enclose various functional molecules that have the potential to foster cell survival [38,39]. For instance, intravitreally administered EVs are as effective as MSCs in improving vision in retinal laser injury models [40]. Mead and Tomarev demonstrated that EVs from the MSC-protected retinal ganglion cell function in a rat optic nerve crush model [41]. In this review, we present a comprehensive overview of the role of PCs in vascular function and their involvement in the progression of various retinal and other pathologies during diabetes. We will also reveal the therapeutic potential of stem cell-like PCs and their application in experimental models of retinal diseases as well as in clinical trials. Ultimately, we will discuss the emerging hypothesis that advocates the regenerative function of PCs and their potential use in tissue repair.

## 2. Pericytes (PCs) Heterogeneity

### 2.1. Tissue of Origin

**Figure 1:**
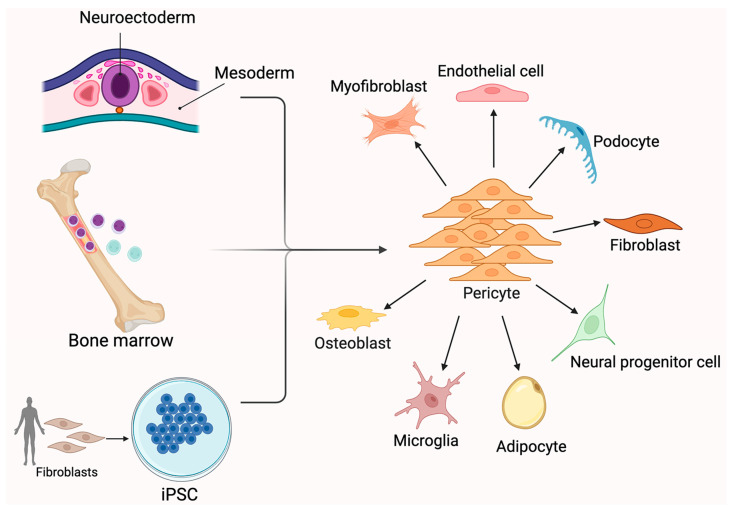
Pericyte sources and differentiation to other cell types. Pericytes can arise from multiple sources, including the neuroectoderm, mesoderm, bone marrow-derived progenitors, and induced pluripotent stem cells (iPSCs). Once differentiated, pericytes serve as perivascular mural cells and exhibit significant plasticity. They can differentiate into a broad range of cell types, such as myofibroblasts, endothelial cells, fibroblasts, podocytes, osteoblasts, microglia, adipocytes, and neural progenitor cells. This versatility contributes to their key roles in vascular stability, tissue repair, and regenerative medicine. Pericyte differentiation is influenced by local microenvironmental cues and disease conditions, highlighting their importance in both homeostasis and pathology. Created in BioRender. Shirbaghaee, Z. (2025) https://BioRender.com/vil3bpc (accessed on 29 May 2025).

The developmental origin of PCs has long been debated. PCs exhibit heterogeneity with respect to their origin, distribution, phenotype, marker expression, and function [7]. Understanding the origins of PCs will have crucial consequences for understanding the formation of specialized vascular networks in various organs during embryonic angiogenesis [42]. Cerebral and retinal PCs exhibit dissimilarities in their embryonic derivations, as described in the previous studies [43]. It has been postulated that the preponderance of PCs in the CNS is derived from both mesoderm-derived MSCs and neuroectoderm-derived neural crest cells, as shown using chicken–quail chimeras [44] and genetic lineage tracing experiments (Figure 1) [42].

While the mesothelium has been implicated as the source of PCs in coelomic organs, such as the lung, liver, and gut [42], recent research on the thymus, retina, choroid, and aortic outflow tract has revealed that PCs can also originate from differentiated neural crest-derived cells [45]. Coronary PCs and vascular SMCs are produced in the heart by the epicardial mesothelium [42]. Furthermore, recent studies have suggested that a subset of PCs in the embryonic brain originates from hematopoietic progenitors [43,46]. For example, recent publications suggest that B cells generated from bone marrow may be the progenitor cell population of PCs. It has also been observed that ECs and fibroblasts can be persuaded to transform into PCs. However, it is widely believed that PCs are capable of undergoing differentiation very similar to MSCs [44]. PC-like cells have recently been isolated from human pluripotent stem cells. PCs, such as MSCs, could transdifferentiate into several cell subtypes in vitro and in vivo, including chondrocytes, adipocytes, osteoblasts, phagocytes, neural progenitors, vascular cells, and microglia [47].

Yamamoto et al. used high-resolution whole-mount imaging and a variety of genetic lineage tracing to demonstrate that myeloid progenitor cells undergo differentiation into a specific subset of PCs within the ectoderm-derived skin and the brain throughout development [48]. PC formation was impaired in mutant mice lacking the myeloid lineage. Similarly, throughout embryogenesis certain CD31^+^ F4/80^+^ macrophages are associated with cerebrovascular PCs [49]. It remains unknown whether PCs from various sources perform distinct functions. In addition, whether PCs of diverse origins survive in adulthood, and contribute to the prevalence of a certain PC subtype is intriguing. The presence of PCs in pathological conditions contributes to tissue-resident progenitors in adults. Specifically, PCs are generated by MSCs in response to radiation therapy [50], and can be derived from mesenchymal tumors, such as bone and soft tissue sarcomas [51]. Furthermore, PCs are generated by glioblastoma stem cells to promote tumor growth [52].

These investigations conclusively demonstrate that the PC origin exhibits heterogeneity in a tissue- and context-specific manner. Additional investigations into the source of a particular PC type may reveal their possible roles in specific pathological conditions [44]. For instance, following brain injury, one group contributes to the creation of scar tissue that separates injured tissue from nearby healthy tissue [52], whereas another group is able to produce new blood vessels [53]. According to the terminology established by the cancer field, Type-1 PCs (PC1) were used to refer to physiological capillary PCs, and Type-2 PCs (PC2) were used to refer to the pathological fraction of these capillary PCs [54,55]. Both groups are present in brain capillaries with luminal diameters less than 10 µm and the same branching patterns [54]. The successful determination of the source of adult tissue PCs requires the establishment of a cell-type-specific inducible Cre recombinase. Additionally, the utilization of inducible lineage-tracing experiments allow for investigating the potential differential contributions of PCs from distinct origins to neovascularization processes in pathological contexts, including tumor angiogenesis and wound regeneration [42].

### 2.2. Tissue-Specific Distribution and Cross-Organ Heterogeneity

PCs are classified according to their morphological variations, which are associated with various modalities of vascular recruitment and anatomical positioning, while liver PCs, also known as hepatic stellate cells (HSCs), reside in the sinusoidal space are loosely and intermittently associated with ECs and possess a unique vitamin A storage capacity. However, PCs of the CNS microvasculature are tightly and continuously invested around the endothelium to support the vascular barrier properties [56]. The CNS microvasculature has the highest PC-to-EC abundance, with PC density and distribution varying among the organs and vascular beds to fulfill tissue-specific requirements [57]. The fact that PCs display more diversity across organs than vascular SMCs is noteworthy [58,59]. With the advancement of RNA sequencing techniques, we are beginning to learn about species-specific differences among tissue specific PCs [60].

The lack of knowledge regarding inter-tissue variations in these two primary categories of mural cell activity remains. This might be due to PCs having higher cell-intrinsic flexibility to modify their molecular repertoire and perform tissue-specific requirements, while vascular SMCs provide a more pervasive function throughout tissues. In contrast to distinct tissue-specific transcriptome alterations, transcription factor expression in mural cells appears to be well conserved across organs, indicating that epigenetic mechanisms play a significant role in mural cell subtypes [58]. It was recently discovered that DNA hypermethylation regulates alpha smooth muscle actin (α-SMA) expression in renal mural cells following ischemia [61]. This suggests that techniques like assays for transposase-accessible chromatin sequencing (ATAC-seq) will be crucial for exploring mural cell characteristics in greater detail [57].

Despite their diverse shapes, PCs are classified into three categories according to their mural placement and morphology: pre-capillary, mid-capillary, and post-capillary [56,62]. PCs with cell bodies in pre-capillary arterioles, microvessel bifurcation sites, and upstream microvessels (i.e., junctional PCs) display a mesh-like, circular staining structure enveloping microvessels. By contrast, mid-capillary PCs, which have somas on straight sections of a capillary segment, exhibit a helical, strand-like staining pattern [63,64,65]. Remarkably, mesh PCs produce α-SMA more frequently, and with higher levels than mid-capillary PCs, suggesting that junctional PCs are more contractile than mid-capillary ones [63,64]. Alarcon-Martinez et al. identified a novel type of PC with unusual morphology [66]. These PCs exhibited fine tubular processes, in addition to primary and secondary processes, as previously observed in vitro in other cell types. Inter-PC tunneling nanotubes are created, arising from the PC soma, and link to the PCs in nearby capillaries. They include organelles, and express α-SMA to control the diameter of linked capillaries via calcium signaling through the nanotubes [67].

### 2.3. Molecular Signatures of PCs

PCs can be characterized using several commonly employed markers, including neural glial antigen 2/chondroitin sulfate proteoglycan 4 (NG2/CSPG4), PDGFRβ, CD13 (aminopeptidase N), CD146, 3G5 ganglioside, desmin, vimentin, regulator of G protein signaling 5 (RGS5), alkaline phosphatase, and α-SMA [7,33,67]. There is a correlation between the expression of NG2 and α-SMA and the specific type of vessel they encompass. Consequently, PCs that surround capillaries are NG2^+^ α-SMA^−^; whereas those encircling venules are NG2^−^ α-SMA^+^. Moreover, PCs surrounding arterioles manifest NG2^+^ α-SMA^+^ properties, although PDGFRβ and CD146 are found everywhere [2]. Thus, PC localization in microvessels has a significant detection criterion. For example, a combination of PDGFRβ, NG2, and α-SMA, along with their blood vessel localization, can be utilized for retinal PC identification [68]. Additional markers have recently been proposed to detect PCs, including thromboxane 18 (Tbx18) and glioma-associated oncogene homolog 1 (Gli1) [69]. In addition to the well-known markers PDGFRβ, RGS5, CSPG4, and ANPEP, a recent single-cell transcriptomic study discovered genes exclusively expressed in brain PCs, including CD248, KCNJ8, ABCC9, S1PR3, and VTN [70]. Furthermore, Kir6.1 (KCNJ8) displays a significant level of expression within a particular subgroup of brain PCs, but not in others [71].

**Figure 2:**
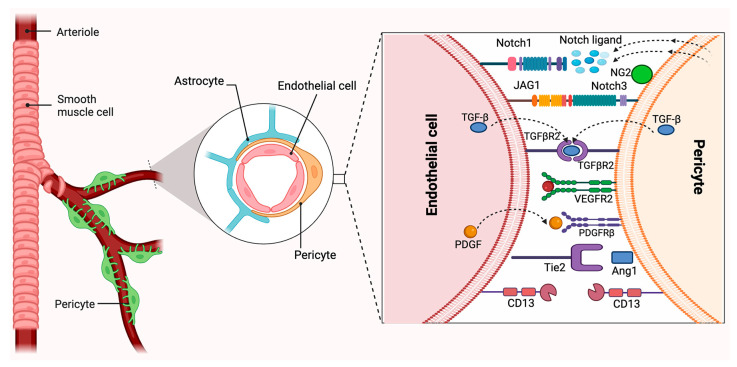
Neurovascular unit and pericyte–endothelial cell–cell interactions. Pericytes are heterogeneous mural cells embedded within the microvascular basement membrane and can be identified by the expression of several markers, such as PDGFRβ, NG2, CD13, VEGFR, and Ang1. Created in BioRender. Shirbaghaee, Z. (2025) https://BioRender.com/b6xom6p (accessed on 29 May 2025).

Other cell types, such as vascular SMCs, perivascular fibroblasts, and macrophages, also express similar markers. During angiogenesis, NG2/CSPG4 is expressed by PCs, yet it is also expressed on glial precursor O2A cells in the CNS, which differentiate into either astrocytes or oligodendrocytes in vitro. PDGFRβ is a well-studied molecular marker expressed in PCs [42]. The expression of PDGFRβ is also not exclusively limited to PCs. This cell surface receptor tyrosine kinase is expressed by a variety of stromal cells, including vascular SMCs and fibroblasts. Berthiaume et al. employed PDGFRβ-Cre/yellow fluorescent protein (YFP) mice, in which all cells produced from PDGFRβ-expressing cells were fluorescently labeled [72]. PCs, vascular SMCs, and ECs may all be identified by CD146, also known as a melanoma cell adhesion molecule (MCAM) [2]. However, PCs do not exhibit EC biomarkers, such as von Willebrand factor and CD31, nor do they exhibit markers of other perivascular cell types, such as glial cells [glial fibrillary acidic protein (GFAP) and OLIG2], microglial cells (IBA1), and neuronal cells [24]. Unfortunately, a definitive and exclusive molecular marker that can accurately identify the entire PC population has yet to be discovered. Significantly, not all cells located in the perivascular sites are PCs. In addition to PCs, additional blood vessel-associated cells, such as microglia, fibroblasts, adventitial cells, macrophages, and vascular SMCs, have also been discovered [7]. Thus, at least two biomarkers among blood vessel anatomical location, cell morphology, and the lack of endothelial and glial cell markers have become necessary for the accurate determination of PCs (Figure 2) [73].

## 3. Pericytes (PCs) and Pathophysiology of Diabetic Microangiopathies and Repair

The role of capillary regression and malfunction (i.e., rarefaction) as primary pathogenic regulators of major human illnesses has become increasingly noticeable [74]. The retina has a higher topographical density of PCs than any other tissue, which provides the optimal integrity of the retinal microvasculature. The human retina has a PC–EC ratio of 1:1, whereas the kidney has a ratio of 1:2.5, the cerebral cortex has a ratio of 1:5, the lungs have a ratio of 1:10, and the skeletal muscle has a ratio of 1:100 [75]. The prevalence of PCs in the retina highlights their role in structural and functional tasks. Retinal vasculature occupancy by PCs is commonly considered critical for EC survival and normal vascular function [76]. Reduced retinal PC coverage is associated with increased vascular permeability, pathological neovascularization, macular edema, and non-perfusion as occurs during diabetes. Similarly, PC loss can cause the inner BRB breakdown, contributing to the pathology of diseases, such as uveitis and AMD [77].

**Figure 3:**
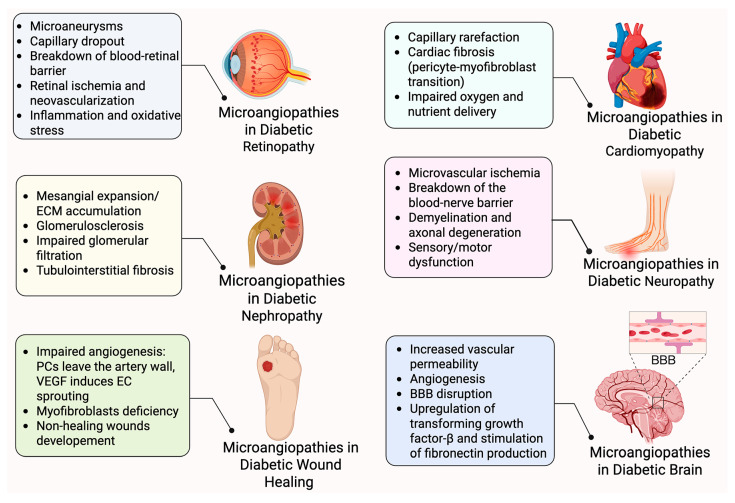
Pericytes and pathophysiology of diabetic microangiopathies. Pericyte loss or dysfunction contributes to diabetes complications across multiple organs. In each tissue, pericyte impairment leads to vascular instability, increased permeability, inflammation, fibrosis, and impaired regeneration. Many organs are affected, including the eye, kidney, heart, nerves, brain and skin, each showing distinct pathological outcomes linked to pericyte-related microangiopathies. Created in BioRender. Shirbaghaee, Z. (2025) https://BioRender.com/hafq4qs (accessed on 29 May 2025).

Many retinal vascular disorders, such as retinopathy of prematurity, DR, retinal vein occlusion, and Norrie disease, are associated with dysregulation of PC–EC communication, which exacerbates disease development. PC–EC miscommunication in retinal neurovascular disorders can be caused by PC and EC loss, as well as harmful environmental factors and abnormal signaling pathways. For example, in preclinical oxygen-induced ischemic retinopathy (OIR) models and preterm infants, elevated oxygen levels promote the obliteration of the developing vasculature, which leads to retinal ischemia and pathological angiogenesis [78]. Vascular injury can also be induced by inflammation and oxidative stress, leading to an elevation in the BRB permeability during ischemia and DR [79]. Norrin/Frizzled 4 (FZD4) signaling is disrupted in Norrie disease, resulting in irregular vasculature and BRB/BBB leakage in the retina and the brain [80]. In the following section, we capture recent data showing new observations that link PC dysfunction and loss in the pathophysiology of retinopathies and repair. Diabetes is a chronic medical disorder, and results in multiple vascular dysfunctions in all organs and tissues, including the brain, heart, kidney, and neurons (Figure 3) [81]. Diabetes impacts on PCs in these organs contribute to various dysfunctions in these tissues and organs [82]. We will examine current studies that suggest structural microvascular abnormalities in these organs preceding microvascular complications and dysfunction. We will also discuss potential treatment approaches that target PCs.

### 3.1. Diabetes Mediated Microangiopathies in the Retina

Degeneration of the retinal microvasculature is a hallmark of DR, which leads to the aberrant formation of retinal blood vessels, elevated capillary permeability, hypoperfusion, ischemia, neovascularization, and subsequent vision loss [83]. Preclinical studies have provided strong evidence that PC loss is a very early step in retinal vascular dysfunction associated with the development and progression of DR [84]. Fundus alterations from patients with diabetes can be used to categorize DR into two main stages: non-proliferative and proliferative [84]. The early stages of DR, in which PCs are the main target of hyperglycemic stress, are characterized by PC damage and death [85]. Since the EC-to-PC ratio increases from 1:1 in healthy retinal structures to 4:1 in diabetic retinal structures, PC loss has been identified as one of the early structural abnormalities in diabetic retinal vasculature [86]. This is attributed, at least in part, to the differential sensitivity and metabolic activity of PCs compared with ECs and astrocytes under high glucose conditions [87].

The non-proliferative stage is limited to the retina. In this stage, blood vessels experience thickening of the basement membrane, vascular instability, microaneurysms formation and bleeding, vascular degeneration, and macular edema. By contrast, the proliferative stage is distinguished by the formation of new abnormal retinal blood vessels or neovascularization. These new blood vessels are susceptible to rupture, which may ultimately result in retinal hemorrhage and detachment [88]. Microaneurysms can develop and increase the risk of retinal hemorrhages [74]. Microaneurysm development is mostly influenced by PC loss [89]. A decrease in blood flow to the retina occurs because of early PC loss, which is quickly followed by EC loss and the collapse of the capillary network. It has been suggested that Angiopoeitin-2 (ANGPT2) is the primary cause of early PC loss [90]. PC detachment and shifting in the microvasculature are caused by external stimuli, including ANGPT2 and PDGFB. However, these effects may be reversed by insulin therapy, demonstrating the dynamic nature of PCs in the blood vessels [85]. In the OIR paradigm, PDGFB signaling via PDGFRβ and the non-catalytic region of the tyrosine kinase adaptor protein (NCK) trigger excessive α-SMA expression and causes pathological neovascularization in PCs enveloping branching blood vessels [91]. As soon as the PC numbers diminish, either in the process of developing new vessels or during adulthood, followed by VEGF addition, the severity of the phenotypes associated with DR is restricted by the inhibition of ANGPT2 activity [16].

Diabetic capillaries feature aberrant basement membranes, most likely due to inappropriate EC and PC interactions. Basement membrane remodeling may be a pathological aspect of this condition, releasing several growth factors, thereby allowing for continuous EC morphogenesis or faster vascular regression. However, the capillary vasculature seems unstable, with aspects of creation and regression occurring concurrently and producing clinically significant outcomes, such as increased retinal blood vessel permeability and hemorrhage, both of which could contribute to vision loss [74]. Thus, the detection and identification of early changes with diabetes could aid in the development of preventive measures against the development and progression of DR.

### 3.2. Diabetes Mediated Microangiopathies in the Brain

The blood–brain barrier (BBB) is an essential structural barrier at the brain parenchyma interface and the circulatory system. It is essential for maintaining homeostasis by controlling communication between the brain and the blood microenvironment [92]. In the NVU, ECs, PCs, microglia, astrocytes, and neurons come together as a functional unit. The impact of inflammation on cerebral blood flow is significant, and poor permeability regulation can result from damage to these cell types, particularly ECs and PCs [93]. The BBB capillaries are also covered by PCs. They are an integral component of the brain vasculature, supporting the development and maintenance of the BBB [94,95].

The impact of diabetes on brain PCs is much less investigated [96]. Severe hyperglycemia exacerbated by histological damage, BBB disruption, brain edema, and PC loss was observed in diabetic mice, ultimately resulting in cognitive impairment [97]. Recent studies have demonstrated that diabetes induces PC loss through elevated oxidative stress [98], thereby reducing vascular coverage in the brain. This reduction is linked to the increased vascular permeability and characteristics of angiogenesis [99]. PC deficiency, driven by increased oxidative stress due to excessive glucose metabolism via the Krebs cycle, could contribute to the BBB disruption [100]. We proposed that the enhanced activity of mitochondrial carbonic anhydrases to support glucose metabolism through the Krebs cycle, promotes PC loss in diabetic mice; inhibiting the carbonic anhydrase activity in PCs reduces oxidative stress and prevents their loss [101].

Advanced glycation end-products (AGEs) stimulate fibronectin production in PCs through the autocrine upregulation of transforming growth factor-β (TGF-β) production [102]. Although the molecular mechanisms behind the BBB leakage of large plasma proteins are not fully understood, potential factors include enhanced endothelial caveolae activity leading to plasma protein transcytosis [103], reduced expression of tight junction proteins, like occludin and claudin-5, and decreased levels of PC-specific markers, such as PDGFRβ (a biomarker for PC damage) and α-SMA. Additionally, increased expression of the inflammatory mediator matrix metalloproteinase 9 (MMP9) has been associated with paracellular permeability [104]. Further research is needed to assess the impact of diabetes on brain PC functions and BBB changes. This knowledge may provide new treatment targets for improving the adverse effects of diabetes on brain functions [105].

### 3.3. Diabetes Mediated Microangiopathies in the Kidney

Diabetic nephropathy (DN) is a common and serious microvascular complication of diabetes that significantly contributes to end-stage renal disease [106]. DN involves various pathological changes in both the glomeruli and the tubular system, leading to disruption of the filtration barrier and the presence of protein in the urine [107]. Hyperglycemia has a significant impact on renal vascular PCs/mesangial cells, and plays a crucial role in the progression of DN. These perivascular supporting cells, which are found around the peritubular and glomeruli capillaries, encircle the nephron vasculature with a PC-to-EC ratio of 1:2.5 [3].

Renal PC-like cells, such as peritubular PCs, mesangial cells, and podocytes, are highly vulnerable to oxidative stress caused by elevated glucose levels, significantly contributing to the progression of DN [5,108]. The key pathological features of DN, including peritubular capillary rarefaction, peritubular fibrosis, mesangial and glomerular hypertrophy, and podocyte damage, are strongly linked to renal dysfunction [5,107,109,110,111]. Mesangial cells are crucial for providing structural support to glomerular capillaries and regulating glomerular filtration. Podocytes are vital for maintaining the integrity of both peritubular capillaries and tubular structures [112]. Additionally, several factors, including glomerular and peritubular capillary rarefaction, loss of PCs, peritubular fibrosis, and arteriosclerosis, collectively contribute to the progressive decline in the renal functions associated with diabetes [111].

Persistent elevated blood glucose levels induce the relocation of peritubular PCs from the capillary into the interstitial space, leading to capillary destabilization and subsequent microvascular rarefaction [113]. Podocytes not only separate from the endothelium, which causes capillary dropout, but they also multiply, migrate, and change into myofibroblasts in response to a variety of stimuli [114]. Pericyte trans-differentiation may be a crucial source of myofibroblasts in fibrotic kidneys, according to new research [113,115,116,117]. Many clinical manifestations of DN are linked to increased matrix production by renal PCs, including peritubular and mesangial cells, triggered by oxidative stress from elevated glucose levels, which activate various signaling pathways, including those involving protein kinase C (PKC) [118,119]. This activation promotes the expression of matrix proteins [120], while suppressing the expression of matrix-degrading genes, like MMPs [121].

Additionally, mitogen-activated protein kinase (MAPK) and extracellular reactive oxygen species (ROS) enhance TGF-β expression in peritubular PCs, podocytes, and mesangial cells [122,123]. TGF-β, a key pro-fibrotic factor in DN, drives the increased production of collagen type IV and fibronectin, while simultaneously inhibiting matrix degradation [122,124]. Lin et al. identified peritubular PCs in the kidney using green fluorescent protein to label collagen-type I-producing cells. These cells expressed classical PC markers, including PDGFRβ, CD44, CD90, NG2, and α-SMA early, but the expression levels diminished as the kidney matured. Following kidney injury, these genes were upregulated in PCs, particularly α-SMA, along with other genes characteristic of the myofibroblast phenotype. This led to an increased production of matrix proteins, notably collagen type I [113].

PCs detach from capillaries and migrate into the interstitial space, where excessive matrix deposition results in tubulointerstitial fibrosis and impaired organ function [113]. According to recent research utilizing single-cell RNA sequencing, myofibroblasts in both men and mice mainly originate from fibroblasts and PCs [125]. High glucose conditions, which cause mesangial cell hypertrophy, may be promoted by increased mTOR activity via downregulation of connexin43 (Cx43). In glomerular mesangial cells, high glucose levels reduce Cx43 expression, inhibit PTEN, and activate the AKT/mTOR signaling pathway. This leads to cell cycle arrest, reduced cell proliferation, and cell hypertrophy. Overexpression of Cx43 counters these effects by restoring PTEN activity and suppressing AKT/mTOR activation, thereby normalizing cell growth and reversing hypertrophy. Conversely, inhibiting Cx43 mimics the effects of high glucose. These findings suggest that Cx43 regulates glucose-induced hypertrophy in glomerular mesangial cells by modulating the PTEN/AKT/mTOR pathway [5,126].

In diabetic rats and mice, glomerular cell loss and apoptosis are commonly observed, correlating with albuminuria and impaired renal function [127,128]. Mechanistically, the elevated urinary level of miR-15b-5p is detected in diabetic patients and db/db mice, contributing to the high glucose-induced apoptosis of mesangial cells [129]. Furthermore, the serum ANGPT2 level is elevated in diabetic individuals and db/db mice, with the ANGPT2/miR-33-5p/SOCS5 signaling pathway playing a role in mesangial cell apoptosis under high glucose conditions [130].

Podocyte loss is an early pathological marker in DN and contributes to proteinuric glomerulopathies [131,132,133]. Apoptosis is the most common mechanism behind podocyte loss, with extensive documentation supporting its role [132,134,135,136]. Mechanistically, ROS, AGEs, microRNAs (miRNAs), and angiotensin II, along with activating pathways like p53, mTOR, and Notch, contribute to DN-induced podocyte apoptosis [132,137,138]. Additional mechanisms, including autophagy, mitotic catastrophe, anoikis, necrosis, and pyroptosis, are also involved in podocyte loss [132]. VEGF-A, mainly produced by podocytes, is crucial for the survival of glomerular ECs. The loss of podocyte-derived VEGF-A leads to EC dysfunction and disruption of the glomerular filtration barrier [139,140,141].

### 3.4. Diabetes Mediated Microangiopathies in the Heart

PCs are the second most prevalent cell type in the ventricles, making up 20% of the total heart cell population [142]. They are essential for regulating blood flow within the myocardium [143,144]. Additionally, PCs play a significant role in the early progression of diabetes-related cardiovascular conditions, including myocardial and interstitial fibrosis [145,146,147]. Tu et al. reported a decline in cardiac PCs and microvascular coverage in diabetic mice [148]. Similarly, a study on myocardial explants from diabetic patients demonstrated a reduction in capillary density and PC loss compared to non-diabetic samples. These myocardial tissues were collected from individuals with end-stage heart failure undergoing transplantation, with and without diabetes mellitus. Notably, overexpressing thymosin beta 4 in diabetic pigs helped prevent cardiac PC loss, suggesting a promising therapeutic strategy for diabetes-related cardiovascular disease [149].

PCs play a crucial role in the heart’s repair processes following myocardial infarction [150], and contribute to the regeneration of injured and dystrophic skeletal muscles [151]. Research has explored cardiac PCs as potential therapeutic targets after injury. Recent studies have identified microvascular PCs in the human ventricular myocardium, revealing that these cells express mesenchymal stem/stromal cell markers, such as CD44, CD73, CD90, and CD105 [152]. Additionally, endothelial-to-mesenchymal transition (EndMT) plays a role in enhancing fibroblast activation and, under diabetic conditions, EndMT expands within the cardiac interstitial space. PCs influence cardiac fibrosis by promoting myofibroblast conversion and by releasing mediators from vascular cells that stimulate fibroblast activation [153].

Lu et al. [154] analyzed single-cell data from individuals with cardiomyopathy and myocardial infarction, revealing that PC marker genes were highly associated with renin secretion, vascular SMC contraction, gap junctions, purine metabolism, and diabetic cardiomyopathy pathways. In patients with cardiomyopathy, PCs exhibited significantly lower regulation of collagen biosynthesis, modulating enzymes, and collagen formation compared to other cell types. The key hub genes involved in PC functions related to cardiomyopathy were COL4A2, COL4A1, and SMAD3, suggesting their potential as biomarkers and targets for therapy development for diabetes-related cardiovascular diseases [154,155]. Multiple studies have shown that sodium–glucose cotransporter-2 (SGLT2) inhibitors not only lower blood glucose levels in diabetic patients but reduce the risk of cardiovascular events. The cardioprotective effects of SGLT2 inhibitors are attributed to several mechanisms, including enhanced myocardial energy metabolism, reduced myocardial oxidative stress and fibrosis [156], and decreased myocardial sodium/hydrogen exchange [157]. These findings suggest that the loss of cardiac PCs is linked to cardiomyopathy, and that SGLT2 inhibitors help mitigate PC loss while improving cardiomyopathy in individuals with type 2 diabetes [148].

### 3.5. Diabetes Mediated Microangiopathies in Peripheral Nerves

Diabetic neuropathy, one of the complications of diabetes, is a type of distal polyneuropathy caused by metabolic and vascular disruptions linked to the disease. It is marked by a progressive “die-back” pattern of neurodegeneration that primarily affects the peripheral nerves [158,159]. In diabetic neuropathy, several molecular pathways and proteins play key roles in disease progression and may serve as therapeutic targets; bradykinin-1 receptor, though minimally expressed in neurons, mediates intracellular calcium mobilization and contributes to sensory neuron sensitization and hyperalgesia. Early absence worsens neuropathy, while delayed antagonism reduces oxidative stress and restores function. The Wnt/β-catenin pathway is crucial for neuronal myelination and is implicated in neuropathic pain. Its upregulation is observed in diabetic peripheral neurons, and the inhibition of this pathway can prevent diabetic neuropathy.

Rho-associated kinase (ROCK) regulates cytoskeleton and cellular behavior. It is overactive in multiple diabetic complications, including neuropathy, where it impedes axonal regeneration. Inhibiting ROCK, such as the use of fasudil, may improve therapeutic outcomes. Overactivation of Notch signaling promotes inflammation and neuropathic pain via glial activation, altered ion channels, and enhanced synaptic transmission. Its inhibition can reduce neurodegeneration in diabetic neuropathy models. A component of the complement system, complement component 3a (C3a), is linked to inflammation and diabetes complications. Elevated C3a correlates with increased diabetic neuropathy risk, possibly by impairing the blood supply to nerve tissues. Doublecortin-like kinase 1 (DCLK1), a microtubule-associated protein supporting neuronal survival and synaptic development, which is understudied in diabetes, regulates Notch signaling and may influence diabetic neuropathy. Growth associated protein 43 (GAP43) is a neural marker for axonal health and regeneration. Its expression drops in later stages of diabetes and serves as an early indicator of neurodegeneration in diabetic neuropathy [160].

The blood–nerve barrier (BNB) is a functionally dynamic, multicellular vascular structure composed of more than just specialized endoneurial ECs. It also includes PCs, perineurial cells, Schwann cells, the basement membrane, and the surrounding axons, all working together to maintain its integrity and function [161]. Sauer et al. [161] showed neuropathy-induced allodynia and hypersensitivity are accompanied by a loss of PCs in the spinal cord. These results provide evidence that neuropathy opens the BNB for small and larger molecules that are associated with or caused by a loss of PCs [161]. PCs, as a key element of the NVU, support the regenerative capacity of both the central and peripheral nervous systems through their involvement in regulating blood flow, promoting angiogenesis, preserving the BBB, and facilitating neurogenesis and neuroprotection [47].

Studies using cell lines that form the BNB have shown that a PC culture supernatant enhances the BNB integrity by increasing trans-endothelial electrical resistance and reducing the permeability of radiolabeled inulin in BNB-derived ECs. This effect is associated with the elevated expression of tight junction proteins, such as claudin-5 and occludin, in these ECs. PCs secrete several bioactive factors, including ANGPT1, VEGF, basic fibroblast growth factor (bFGF), and glial cell line-derived neurotrophic factor (GDNF). Among these, ANGPT1 and VEGF were found to reduce claudin-5 expression, potentially weakening the blood–nerve barrier function, while bFGF and GDNF increased claudin-5 levels, thereby supporting the barrier integrity. Additionally, PCs contribute to the structural stability of the blood–nerve barrier by producing key basement membrane proteins, such as fibronectin and type IV collagen, along with the tissue inhibitor of metalloproteinase-1 (TIMP-1), which prevents basement membrane degradation. These findings underscore the essential role of PCs in regulating and maintaining both the functional and structural integrity of the blood–nerve barrier [162].

Hyperglycemia-induced apoptosis of PCs contributes to the development of neuropathy and erectile dysfunction in diabetic patients. A reduced number of PCs leads to the disorganization of ECs, resulting in the decreased perfusion of peripheral nerves and consequent hypoxia. The hypoxia is, in turn, responsible for the oxidative stress and apoptotic cell death of PCs, which results in microangiopathies of the endoneurial capillaries. Similarly, a decrease in penile PC number, associated with an increase in corpus cavernosum sinusoidal permeability and erectile dysfunction, was observed in animal models of diabetes mellitus type 1 [76].

### 3.6. Diabetes Mediated Microangiopathies in Wound Healing

Wound healing involves several steps, including inflammation, cell proliferation, migration, angiogenesis, and re-epithelialization [163] that is compromised in diabetic patients, mostly due to diabetes-associated angiopathy. During the proliferation phase, ECs form extensive microvascular networks in the granulation tissue by angiogenesis, which is organized by a fibroblast-deposited ECM [164]. Consequently, excess blood vessels are remodeled and undergo regression to become functional during the maturation phase, thereby repairing wounded tissue. Therefore, impaired angiogenesis results in the development of chronic and non-healing wounds, attributed to various pathological conditions, such as diabetes, peripheral arterial diseases, and high blood pressure [165].

VEGF, a critical biochemical agent essential for the development and control of wound angiogenesis, is released from hypoxic tissue and activates dormant ECs, hence initiating angiogenesis. When tissues are wounded, VEGF-stimulated ECs generate ANGPT2, which causes PCs to leave the artery wall, allowing for VEGF-induced EC sprouting. However, the mechanisms by which ECs and PCs work in concert to generate functional blood vessels during wound angiogenesis remain elusive [166]. The loss and dysfunction of PCs in diabetes severely impede wound healing due to their essential functions in stabilizing both existing and newly created capillaries [167].

Tissue repair begins with granulation tissue formation, where macrophages play an anti-inflammatory role and support healing. However, their involvement in fibrosis and myofibroblast differentiation remains unclear. Myofibroblasts, primarily derived from PCs in organs, like the lungs and liver, are essential for wound closure, and their deficiency leads to chronic wounds and persistent inflammation. Studies have suggested that macrophage-derived amphiregulin promotes wound healing by triggering PC differentiation into myofibroblasts. Amphiregulin binds epidermal growth factor receptor (EGFR) on PCs, activating integrin αvβ3, which stimulates the TGF-β signaling pathway, driving myofibroblast formation. These myofibroblasts then secrete ECM components to enhance tissue repair [168].

## 4. Pericytes (PCs) in Targeted Therapies for Diabetes Microangiopathies

**Figure 4:**
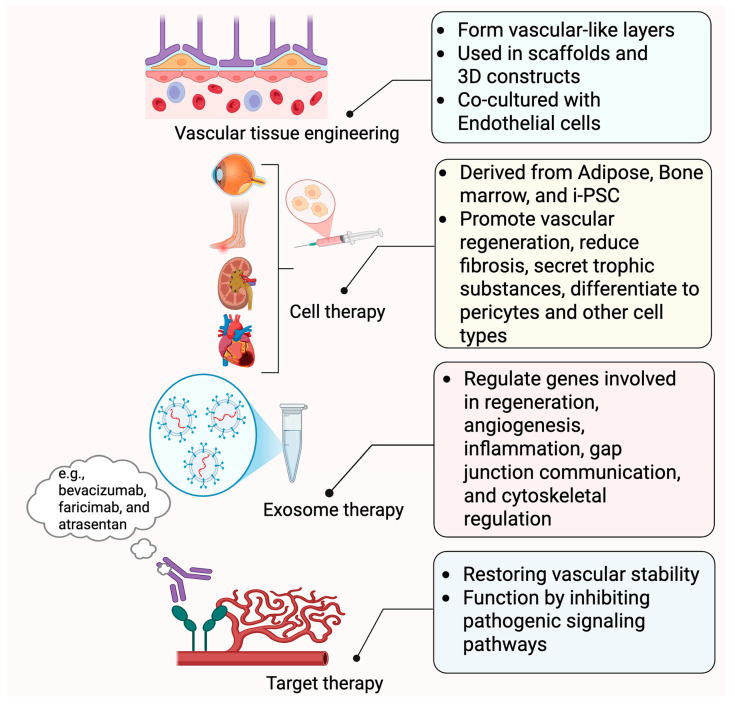
Treatment application of pericytes. This figure summarizes the emerging applications of pericytes in regenerative medicine and vascular therapy. In vascular tissue engineering, pericytes are used to form vascular-like layers within 3D constructs, often co-cultured with endothelial cells. Cell therapies utilize pericyte-like cells derived from adipose tissue, bone marrow, or iPSCs to promote vascular repair, reduce fibrosis, and secrete trophic factors. Exosome therapy leverages pericyte-derived extracellular vesicles to regulate genes related to regeneration, angiogenesis, and inflammation. Finally, targeted therapies aim to restore vascular stability by inhibiting pathological signaling pathways involved in diabetes microvascular complications. Created in BioRender. Shirbaghaee, Z. (2025) https://BioRender.com/grdr2q1 (accessed on 29 May 2025).

As discussed above, PC dysfunction is a key factor in diabetes microangiopathies, including retinopathy, nephropathy, cardiovascular dysfunction, neuropathies, and wound healing, making targeted therapies essential for restoring vascular stability and function by inhibiting pathogenic signaling pathways. The VEGF/VEGFR pathways play crucial roles in angiogenesis, with anti-VEGF treatments, like bevacizumab and faricimab, helping to prevent PC loss and stabilize microvascular networks. Endothelin-1 (ET1) signaling contributes to PC dysfunction in DR, and ET(A) receptor blockers, like atrasentan, reduce PC loss, and iptakalim prevents hypercontractility. PDGFB/PDGFRβ signaling, essential for PC survival and migration, has been explored for vascular stabilization using monoclonal antibodies. Emerging therapies, including adenovirus-based approaches and gene-editing techniques, such as Bim inactivation, offer promising strategies to restore PC function and prevent vascular degeneration in ischemic conditions [169]. Alternatively, the use of PCs to replace damaged cells could restore normal vascular functions (Figure 4).

### 4.1. PCs in Cellular Therapy

PCs are essential for vascular remodeling and stability, making them promising targets for cellular therapy. They share similarities with mesenchymal and adipose-derived stem cells, suggesting potential therapeutic applications. Disruptions in PC–EC interactions contribute to diseases like DR, fibrosis, and cancer, underscoring the need for targeted therapies [25]. PC transplantation has shown promise in muscle, heart, and brain diseases, though its application in retinal vascular diseases remains in the early stages. Harnessing PCs from stem cell sources could enable therapies that promote vascular stabilization and regeneration, making a deeper understanding of PC biology crucial for advancing regenerative medicine.

Stem cell-based treatments, particularly MSC transplantation, have shown promise in treating retinal disorders via two basic mechanisms: secretion of trophic substances that help local cells survive, and differentiation of PCs or protection against PC loss [169]. Adipose tissue-derived MSCs produce neurotrophic factors, such as brain-derived neurotrophic factor (BDNF), nerve growth factor (NGF), and GDNF, which promote neurogenesis and protect retinal cells. Furthermore, miRNA-192 and miRNA-222 reduce inflammation and promote retinal vascularization. Experimental data from OIR and DR models show that transplanting adipose-derived stem cells expressing PC markers protects against vascular degeneration and stimulates vessel regeneration [170,171].

PC transplantation has demonstrated therapeutic efficacy in various organ systems. In muscle repair, intramuscular injection of 5 × 10^4^ PCs derived from mouse skeletal muscle into a hindlimb of C57BL/6J mice successfully regenerated myofibers in injured mouse muscles. Notably, CD146+ PCs exhibited superior regenerative capacity, enhancing capillary density and collagen turnover more effectively than NG2+ PCs. The results suggest that the selection of PCs based on CD146 rather than NG2 results in the isolation of therapeutic mural cells with a high capacity to positively remodel skeletal muscle after a period of immobilization [172]. In cardiac regeneration, PC progenitor cells from saphenous veins transplanted into infarcted mouse hearts reduced myocardial scarring, interstitial fibrosis, and pathological neovascularization, improving vascular permeability and cardiac function. These benefits were largely attributed to PC-derived miR-132, which modulates vascular remodeling [150].

In brain repair, due to structural similarities between the CNS and the retina, PC transplantation has been investigated in stroke and neurodegenerative diseases. In a murine model of transient middle cerebral artery occlusion, the intravenous transplantation of 1×10^6^ mouse pericyte-like cells generated from human pluripotent stem cells (hPSC) through the intermediate stage of the cranial neural crest (CNC) maintained the BBB integrity, reduced neuronal apoptosis, and promoted neurological recovery. thereby revealing that cells expressing typical PC markers, including PDGFRβ, CD146, NG2, CD13, caldesmon, and vimentin, display distinct contractile properties, vasculogenic potential, and endothelial barrier function. Additionally, MSC-derived PCs in Alzheimer’s models improved microcirculation and amyloid clearance, demonstrating their neuroprotective potential [173]. Agafonova et al. [174] demonstrated that PC-like adipose-derived stem cells exhibit enhanced PDGFRβ expression, nuclear Nrf2 localization, and increased HO1 protein content when co-cultured with human retinal ECs under hyperglycemic conditions. Additionally, these co-cultures showed reduced cytosolic phospholipase A2 activity, prostaglandin E2 release, and VEGF levels. These findings suggest that autologous PC-like adipose-derived stem cells may offer a promising cell therapy approach to mitigate DR-induced vascular damage [174].

### 4.2. PCs in Regeneration and Wound Healing

PCs behave as MSCs and play a key role in regeneration across various tissues, including muscle, fat, and nerves. They contribute to angiogenesis by interacting with ECs through signaling molecules like VEGF, TGF-β, PDGFRβ, and angiopoietins [175]. Recent findings have suggested a lineage connection between PCs and mesenchymal or adipose-derived stem cells, positioning them as potential mediators of vascular remodeling. However, their close association with progenitor cells complicates their precise identity. Understanding the molecular mechanisms governing PC–EC interactions is essential for developing cell-based therapies, offering new intervention strategies for vascular-related diseases [25].

Kim et al. [175] investigated the role of PCs in angiogenesis and wound healing under high glucose conditions using in vivo and in vitro models. Co-culturing PCs with human umbilical vein endothelial cells (HUVEC) enhanced tube formation and cell migration. In addition, the injection of a collagen type I scaffold with 5 × 10^5^ PCs in the wound in a diabetic mouse model showed improved wound healing through increased vascularization. These findings suggest PC transplantation as a potential therapy for ischemic foot ulcers [175]. For patients suffering from severe limb ischemia, who are not candidates for endovascular angioplasty or bypass surgery, cell-based therapeutic angiogenesis is a crucial strategy to enhance blood flow to the affected areas. MSCs are currently viewed as one of the most promising regenerative options, aiding tissue repair and functional recovery through their roles in angiogenesis and immune modulation. Encouraging outcomes from preclinical studies have led to numerous clinical trials aimed at evaluating the safety and efficacy of MSCs derived from various sources for treating limb ischemia. Further details on these clinical trials can be found at the ClinicalTrials.gov website (www.clinicaltrials.gov; accessed 15 April 2025) [176].

Regenerative treatments for limb ischemia based on MSC therapy are still considered experimental. MSC injections into rat eyes improved retinal functions, reduced apoptotic cells surrounding the retina, and decreased vascular leakage. In vivo, MSCs develop into PCs, with both functional and phenotypic features, indicating that they have the potential to restore retinal vasculature integrity. They also ameliorate ischemia, increase angiogenesis, and provide protection against nerve injury by direct contact with the endothelium lining or through paracrine agents [177]. Rampin et al. [178] developed a protocol to create ECM-rich, autologous PC cell sheets for therapeutic delivery. PCs were isolated from the skeletal muscle biopsies of both diabetic foot ulcer patients and non-diabetic individuals. PCs from diabetic foot ulcer patients successfully produced an ECM, supporting their use in cell-based therapies for limb ischemia. The ECM deposited by the diabetic PCs was also compared to that from the non-diabetic PCs to evaluate differences in the basement membrane components, aiming to optimize future clinical applications [178]. Given the significant benefits of stem cell-based therapy for DR and other eye diseases, human clinical trials are underway using adipose-derived stem cells, bone marrow stem cells, and iPSCs [177].

Several randomized clinical trials (NCT04136743, NCT03379168, NCT04274543, NCT04929951, NCT03467919, NCT06121882, and NCT05660772) are underway exploring the effect of autologous adipose tissue injections for knee osteoarthritis and spinal cord injury. Rich in bioactive elements and regenerative perivascular cells, like PCs, adipose tissue provides cushioning, fills structural defects, and retains mesenchymal stromal cells within an intact stromal vascular niche, making it suitable for clinical applications. Adipose tissue is obtained from the lower flanks bilaterally and the abdomen. The lipoaspirate is then transferred to the Lipogems^®^ device (Lipogems International, Milan, Italy) to wash and mechanically break it down for injection. The final product consists of micronized fat tissue yielding fat clusters with preserved vascular stroma of about 500 microns with intact stromal vascular niche harboring regenerative cellular elements. The resulting product contains PCs, retained within an intact stromal vascular niche, that is ready to interact with the recipient tissue after transplantation and becoming activated as MSCs. Such PC-to-MSC activation entails the release of regenerative factors, causing the transplanted Lipogems^®^ product to act as a “time-release medium” of these factors where they are most needed. One purpose of these studies is to explore the efficacy of autologous, micro-fragmented adipose tissue (Lipogems^®^) injections under ultrasound guidance for chronic shoulder pain in persons with spinal cord injury compared with the standard-of-care, corticosteroid injection [179,180,181].

Bone marrow-derived mesenchymal stem cells (BM-MSCs) are another cell population of interest that have been used in several clinical studies. In one clinical trial (NCT01736059), an average of 3.4 million autologous CD34⁺ BM-MSCs were delivered intravitreally to patients with permanent vision loss caused by retinal vascular diseases. No adverse local or systemic effects were observed associated with this cellular therapy, including in DR [182,183].

Similarly, clinical trials NCT03011541, NCT01920867, and NCT02280135 are also investigating the use of autologous BM-MSCs for treating DR and related retinal conditions. These trials consist of three arms using retrobulbar, subtenon, intravitreal, subretinal, or intravenous injections. Arm one used stem cell concentrates provided by retrobulbar and subtenon injections, followed by intravenous injections. Arm two used stem cell concentrate provided by retrobulbar, subtenon, and intravitreal injections, followed by intravenous injections. Arm three treated the eye with better visual acuity than using Arm one or Arm two, and the eyes with more severe visual acuity impairment received a core pars plana vitrectomy, followed by subretinal or intraoptic nerve stem cell concentrate injections. BM-MSCs are provided through retrobulbar, subtenon, and intravenous injections for one or both eyes. The improvement or stabilization of vision was found to occur for the vast majority of reported patients, and the findings were highly statistically significant [184,185,186,187,188,189,190,191,192,193].

The homing efficiency of diabetic endothelial progenitor cells (EPC) can be enhanced with nitric oxide treatment. Researchers generated vascular progenitors from CD34+ cord blood-derived iPSCs, treating them on fibronectin with VEGF. Selected CD31^+^ CD146^+^ cells, when injected into the vitreous of NOD/SCID mice (an ischemic retina model), homed to acellular capillaries as PCs or engrafted as ECs when delivered intravenously. Hypoxia-induced SDF1 and VEGF guided their migration. Future research aims to repopulate acellular capillaries in diabetic models using these iPSC-derived vascular progenitors, advancing autologous regenerative medicine for vascular repair [194]. An observational clinical trial (NCT03403699) is exploring the use of iPSC-derived ECs and PCs to treat DR. Since, in diabetes, vascular progenitor cells, such as endothelial colony-forming cells (ECFCs), are depleted, and bone marrow-derived progenitor cells are dysfunctional, this trial tested the ability of hiPSC-derived ECFCs to form durable blood vessels in vivo and aid in therapeutic revascularization by integrating with the host rodent vessels. A specific subset of mesoderm cells (SSEA5-KNA^+^) from hiPSCs was found to generate ECFCs. When these cells were combined with CD34^+^ and CD45^+^ cells, the mixture enhanced ECFC survival, function, and repair by reducing the retinal oxidative stress and inflammation. The study hypothesized that hiPSC-derived mesodermal cells and co-administration with CD34^+^ and CD45^+^ cells could provide long-term revascularization and therapeutic effects for DR and macular ischemia. The trial aims to further investigate the potential of iPSCs in regenerating vascular cells in damaged capillaries and enhancing vessel formation in diabetic eyes (https://clinicaltrials.gov/search?cond=NCT03403699 (accessed on 29 May 2025)).

### 4.3. PCs in Tissue Engineering and Restoration of Normal Vasculature

PCs play a critical role in vascular tissue engineering through supporting vascular regeneration by improving the functionality of engineered grafts. When seeded onto biodegradable scaffolds, PCs enhance graft patency and vascular integration, making them valuable for regenerative medicine. PCs appear to maintain their phenotype within synthetic polymer scaffolds and contribute to vessel formation after implantation. In biphasic vascular models, PCs form the media layer, supporting ECs in the intima, and enabling the development of complex, layered tissues, like blood vessels, skin, or myocardium. Furthermore, combining human umbilical cord vein PCs (especially CD146+ cells) with natural ECM components (e.g., collagen type I, fibrin, and elastin) enables the creation of triple-layered, small-diameter vascular constructs. These natural constructs closely mimic native blood vessels and represent a promising step toward clinical-grade vascular grafts for treating conditions like coronary artery disease [195].

To achieve effective vascularization in tissue-engineered grafts, scaffolds must be evenly vascularized to ensure the adequate perfusion and viability of the inner regions. Three main strategies have been developed to support this goal. These include integrating pro-angiogenic cells into the scaffold, designing the scaffold to mimic vascular-like architectures that guide new vessel formation, and incorporating angiogenic growth factors during the fabrication process. Ultimately the aim is to produce grafts with newly formed blood vessels that are both mature and functionally effective [196]. Induced pluripotent stem cell-derived vascular cells—including vascular SMCs, ECs, and PCs—can be employed to construct 3D vascular models, like tissue-engineered blood vessels, tissue-engineered vascular grafts, organoids, and vessel-on-a-chip systems. These models are cultured within bioreactors or chip platforms to apply mechanical stimuli, such as shear stress and cyclic stretch, promoting maturation and functionality similar to native vessels. These advanced systems serve applications in drug testing and development, and tissue-engineered blood vessels may also be utilized as vascular grafts in clinical procedures [197].

In a previous study utilizing multichannel microfluidic platforms, the researchers compared the vascular-supporting capabilities of PCs and unselected stromal cells. The findings demonstrated that PCs significantly enhanced both angiogenesis and vasculogenesis more than stromal cells. Unlike stromal cells, PCs were closely associated with endothelial vessels as mural cells. A gene expression analysis showed that PC–EC co-cultures upregulated vascularization-related genes, while stromal cell co-cultures increased metabolic activity and reduced inflammation. This study highlighted the superior vascular-supporting role of PCs and their mechanisms, with implications for vascular biology and tissue engineering [198].

Using cell tissue engineering techniques, researchers have successfully generated fiber-shaped vascular cell aggregates composed of HUVEC and MSCs by seeding the cells into silicone culture grooves equipped with aggregate stoppers to anchor them to the culture surface. After one week of culture, the cells self-organize into an inverted, vessel-like structure featuring a distinct arrangement. An outer endothelial layer formed by HUVEC, a supporting MSC layer beneath it, and centrally located capillary-like structures made of HUVEC [199].

A scalable 3D bioprinted model of kidney fibrosis was developed to support the development of anti-fibrotic therapies for chronic kidney disease (CKD). Researchers created human cell lines representing kidney PCs (PDGFRβ+), EPC (CD10+), and ECs (CD31+), and confirmed their similarity to native kidney cells using bulk RNA sequencing. The PCs could differentiate into myofibroblasts when exposed to TGFβ. Using a fibrinogen/gelatin-based hydrogel, the team successfully bioprinted these cells into a model mimicking the kidney tubulo-interstitial environment. The model reproduced key mechanisms of kidney fibrosis, such as EC–PC crosstalk and myofibroblast activation following epithelial injury. Importantly, the system is scalable to a 96-well format, making it suitable for drug testing and therapeutic screening [200].

A 3D triculture model of the NVU was developed using human brain microvascular ECs, astrocytes, and PCs, enabling long-term in vitro studies. This model replicates the complex cellular interactions of the NVU and is used to study disease mechanisms. When subjected to mechanical injury mimicking traumatic brain injury, the model exhibits increased cell death, inflammation [e.g., elevated lactate dehydrogenase (LDH), tumor necrosis factor (TNF)-α, monocyte chemoattractant protein (MCP)-2, and MCP-3], and reduced tight junction protein zona occludin (ZO)-1 expression. This system provides a valuable platform for exploring traumatic brain injury-related damage and immune responses within the NVU [201].

### 4.4. MSC-Derived Extracellular Vesicles (EVs) in Treatment of Microangiopathies

In terms of therapeutic applications, the secretome of MSCs is now the most extensively studied. However, other cell types, such as PCs, may have comparable qualities to MSCs, and their potential therapeutic applications are only beginning to emerge. PCs release a heterogeneous secretome of pro- and anti-inflammatory molecules, pleiotropic cytokines, and several stem cell chemokines in response to various stimuli in vitro, implying that PCs may be an underutilized target cell for modulating inflammatory responses to pathogenic stimuli [202]. Exosomes, nanosized EVs, have shown remarkable potential in tissue regeneration and damage healing when compared to their parent stem cells. Exosomes serve an important function in cell communication by delivering biological molecules to locations where they are less immunogenic and can penetrate the BBB [203]. MSC-derived exosomes are being investigated as a potential new source of protective and regenerative ingredients in neuroinflammatory and neurological diseases. However, it is unclear if PCs produce exosomes with features comparable to MSCs [202].

Exosome-based therapies have shown promise in treating spinal cord injury by enhancing vascular stability and reducing inflammation. A study using a rat model demonstrated that bone marrow-derived MSC-exosomes (BMSC-EXOs) protected PCs, reduced neuronal cell death, preserved myelin structure, and improved the blood–spinal cord barrier integrity. Additionally, BMSC-EXOs decreased caspase-1 expression and interleukin-1β (IL-1β) release, reducing inflammation and promoting motor function recovery [204]. Another study highlighted the role of PC-derived exosomal miR-210 in protecting vascular ECs by inhibiting lipid peroxidation and preserving mitochondrial function. This process enhanced the BBB integrity via the JAK1/STAT3 pathway, suggesting that miR-210-5p plays a crucial role in restoring EC functions after spinal cord injury [205].

PC-derived EVs play an essential regulatory role in neurovascular health. They support the formation of new blood vessels (angiogenesis), help preserve the integrity of the blood–tissue barrier and offer protective effects to nerve cells. These vesicles carry a range of bioactive molecules, including growth factors, like VEGF, connective tissue growth factor (CTGF), bFGF, ANGPT1, and neurotrophic factors, such as BDNF, NGF, and GDNF. Additionally, they transport cytokines, including interleukins IL-6, IL-8, IL-10, and MCP-1. PC-derived EVs also contain miRNAs and circular RNAs associated with neurovascular function and the development of various vascular and neurological disorders. As such, therapies based on PC-derived EVs show promise for treating both vascular and neurodegenerative diseases [47].

MSCs exert their therapeutic effects through paracrine signaling, secreting exosomes containing RNAs, lipids, and bioactive molecules. These MSC-EXOs have demonstrated regenerative potential across multiple organ systems. In DN, podocyte apoptosis and VEGF dysregulation contribute to disease progression. Exosomes from human urine stem cells carrying miR-16-5p, helped prevent podocyte apoptosis, maintain the glomerular filtration barrier, and regulate VEGF expression. Additionally, MSC-EXOs from various sources, including bone marrow, adipose tissue, umbilical cord, and amniotic fluid, have renoprotective effects in DN by enhancing autophagy, reducing renal fibrosis, and modulating inflammation [206].

One study highlighted the therapeutic potential of exosomes derived from PC-like immortalized adipose-derived MSCs in protecting retinal microvascular ECs from high glucose-induced damage, a key factor in early DR. These exosomes were efficiently internalized, reducing apoptosis, oxidative stress, and inflammation, while preserving EC barrier integrity and normalizing pro-angiogenic and pro-inflammatory markers. A molecular analysis revealed that exosome treatment regulated the genes involved in angiogenesis (VEGF-A, ANGPT2, and MMP9), inflammation (IL-1β and TNF-α), gap junction communication (CX43), and cytoskeletal regulation (ROCK1) and stability. These findings suggest that PC-like cell-derived exosomes offer a promising strategy for early DR intervention by restoring EC function and vascular stability [207].

Preclinical studies have demonstrated the therapeutic potential of MSC-EXOs in various nervous system disorders, particularly diabetic peripheral neuropathy [208,209,210]. MSC-EXOs suppress inflammation, promote neurovascular remodeling, and enhance functional recovery in diabetic mice. Exosomes were extracted from the culture medium of mouse MSCs using ultracentrifugation. Diabetic db/db, 20-week-old mice served as models for diabetic peripheral neuropathy. Fluorescently labeled MSC-derived exosomes were administered through the tail vein once a week for 8 weeks (1 × 10⁹ particles per animal). The MSC-EXO therapy reduced the markers of M1 (pro-inflammatory) macrophages and elevated those associated with M2 (anti-inflammatory) macrophages. Additionally, the treatment led to a notable reduction in pro-inflammatory cytokine levels. A bioinformatic analysis indicated that the exosomes were rich in miRNAs targeting the TLR4/NF-κB signaling pathway [211]. Additionally, in a rat model of streptozotocin-induced diabetes, rat BM-MSC-derived exosomes [100 μg/kg/dose suspended in 0.2 mL phosphate-buffered saline (PBS)] alleviated diabetic peripheral neuropathy by inducing autophagy through the mTOR signaling pathway [212]. Moreover, exosomes delivering miR-146a significantly enhanced the therapeutic effects of MSCs in diabetic db/db mice, suggesting a promising strategy for diabetic peripheral neuropathy treatment [213]. Further studies are needed to explore the protein and miRNA composition of PC-derived exosomes and their potential function in retinopathies and regeneration of other tissues and organs affected by diabetes.

### 4.5. Challenges in MSC Therapies

While MSC transplantation has produced encouraging outcomes in animal models, there are still significant challenges for clinical translation. Xenogeneic incompatibility is a key challenge, demanding optimal human-compatible models. Standardized transplantation procedures must be created to assure safety, effectiveness, and repeatability [169]. On the other hand, the regeneration capacity of stem cells may be influenced by the donor’s health status, as demonstrated in MSC research. For example, BM-MSCs from rats with chronic kidney disease exhibited decreased proliferation and no therapeutic efficacy due to early cellular senescence [214]. Similarly, BM-MSCs from aging people and Parkinson’s disease patients demonstrated mitochondrial dysfunction, and those from multiple myeloma patients had poor osteogenic differentiation [215,216,217,218]. An et al. [219] investigated perivascular stem cells from both healthy and gestational diabetes mellitus pregnancies, suggesting that maternal metabolic dysregulation during gestation can alter the function of stem cells, which may impact their therapeutic effectiveness. These studies underscore the importance of donor health in the therapeutic use of perivascular stem cell [176,219].

## 5. Conclusions

PCs have emerged as pivotal players in vascular homeostasis and tissue regeneration, particularly in the context of diabetic microvascular complications. Their dual role as vascular support cells and stem-like mediators of repair highlights their therapeutic potential across multiple organ systems affected by diabetes. The growing understanding of PC biology, combined with advancements in tissue engineering and stem cell technologies, paves the way for innovative strategies in regenerative medicine. However, challenges remain in fully characterizing PC subpopulations, standardizing isolation protocols, and translating experimental findings into clinically effective therapies. Continued research is essential to harness the full regenerative capacity of PCs, and to develop targeted interventions aimed at restoring vascular integrity and function in the various organs impacted by diabetes.

## Data Availability

No data was used in the studies presented.
